# High-avidity binding drives nucleation of amyloidogenic transthyretin monomer

**DOI:** 10.1172/jci.insight.150131

**Published:** 2022-04-08

**Authors:** Li Gao, Xinfang Xie, Pan Liu, Jing Jin

**Affiliations:** 1 Feinberg Cardiovascular and Renal Research Institute, Northwestern University, Feinberg School of Medicine, Chicago, Illinois, USA.; 2 Department of Cardiology, and; 3 Department of Nephrology, The First Affiliated Hospital of Xi’an Jiaotong University, Xi’an, China.

**Keywords:** Aging, Cardiology, Cardiovascular disease, Protein misfolding

## Abstract

Amyloidosis involves stepwise growth of fibrils assembled from soluble precursors. Transthyretin (TTR) naturally folds into a stable tetramer, whereas conditions and mutations that foster aberrant monomer formations facilitate TTR oligomeric aggregation and subsequent fibril extension. We investigated the early assembly of oligomers by WT TTR compared with its V30M and V122I variants. We monitored time-dependent redistribution among monomer, dimer, tetramer, and oligomer contents in the presence and absence of multimeric TTR seeds. The seeds were artificially constructed recombinant multimers that contained 20–40 TTR subunits via engineered biotin-streptavidin (SA) interactions. As expected, these multimer seeds rapidly nucleated TTR monomers into larger complexes, while having less effect on dimers and tetramers. In vivo, SA-induced multimers formed TTR-like deposits in the heart and the kidney following i.v. injection in mice. While all 3 variants prominently deposited glomerulus in the kidney, only V30M resulted in extensive deposition in the heart. The cardiac TTR deposits varied in size and shape and were localized in the intermyofibrillar space along the capillaries. These results are consistent with the notion of monomeric TTR engaging in high-avidity interactions with tissue amyloids. Our multimeric induction approach provides a model for studying the initiation of TTR deposition in the heart.

## Graphical abstract

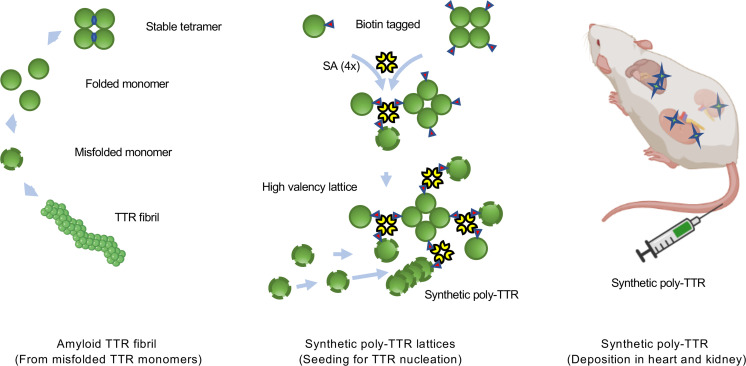

## Introduction

Transthyretin amyloidosis (ATTR) is a systemic disease caused by tissue deposition of TTR fibrils. TTR is an abundant plasma protein mainly produced by the liver. The best characterized function of TTR is as a thyroxine-4 hormone and retinol-binding protein transporter. ATTR deposits of WT TTR are associated with acquired/senile amyloidosis that mainly affects the heart. There are many genetic variants of TTR that cause hereditary ATTR (hATTR) with amyloid deposits in virtually every tissue of the body, most prominently affecting the nervous system, the heart, the eye, the gastrointestinal system, and the vasculature of the brain. Most known hATTR cases are inherited in an autosomal dominant manner. Depending on the individual amino acid substitutions, the clinical course of hATTR varies in terms of onset age and specific tissue and organ involvements. For example, V30M (also termed *TTR*V30M) is the most common hATTR variant that causes TTR familial amyloid polyneuropathy (ATTR-FAP). *TTR*V30M is prevalent among the Caucasian population, particularly in Portugal and Sweden (1). Meanwhile, V122I (*TTR*V122I) is another common genetic variant of hATTR, and its carriers have elevated risks of heart failure due to cardiomyopathy (ATTR-CM) among individuals of African or Hispanic ancestry (2, 3). More than 140 *TTR* variants have been identified with different and overlapping spectra of onset age, clinical manifestations, and risks (4).

TTR protein in its native fold exists as a homotetramer with 2 funnel-shaped thyroxine-binding sites at its dimer-dimer interface (5). Drugs that stabilize TTR tetramers have been developed for treatment of ATTR (6, 7). Ex vivo studies demonstrated that most known hATTR mutants, including V30M and V122I, have a higher tendency than their WT counterparts to misfold and form unstable alternatives of monomers and dimers (8, 9). The structure of TTR monomers contains 8 β-strands, forming two 4-stranded anti-parallel sheets, termed DAGH and CBEF. The DAGH sheet has a tendency of conformational changes in forming amyloid fibril (10, 11). Although the exact role of dimeric TTR remains unclear as to whether it is an intermediate of tetrameric to monomeric, or monomer to oligomer, transformation, its genetic background is high in *TTR*V30M. From a structural perspective, it was noted that *TTR*V30M dimer is connected via an aberrant disulfide bridge facilitated by the steric conformation of the mutant (12, 13). Extensive studies investigated the structural basis for TTR destabilization in subsequent formation of amyloid fibrils (14, 15). It seems that each individual pathogenic variant has distinct characteristics to render amyloidogenic potentials. It should also be noted that, WT TTR, either in its full length or cleaved form (16), is present not only in non-hATTR amyloid fibrils but also in familial deposits either with the accompanying TTR variant (17, 18) or onto existing mutant seeds even after liver transplantation (19–21). In domino liver transplantation using explanted livers from hATTR-FAP donors, organ recipients have the risk of developing systemic amyloidosis (22, 23). Factors that accelerate amyloid fibrillogenesis are being investigated in the context of liver transplantation, including older age of domino liver recipients, as well as the potential of undetectable amyloid nuclei to cause disease transmission in recipients (24). These observations illustrate the importance of amyloid self-seeding or cross-seeding between WT and disease variants, accelerating the growth of TTR fibrils (24, 25).

TTR fibrils extracted from tissues are large complexes with thousands of protein subunits, which also contain other amyloid and nonfibrillar constituents including serum amyloid P component and glycosaminoglycans that are present in all types of amyloids. As unfolded monomeric and dimeric TTRs are prone to form the initial oligomers, these small TTR oligomers are cytotoxic once seeded in tissues with a tendency to nucleate misfolded TTR forms from blood (26–31). Amyloid seeding has been widely studied in many disease types of amyloidosis beyond ATTR (32–34). Regarding TTR, compelling evidence for its amyloid seeding effects came from the observation of patients with hATTRV30M polyneuropathy who had undergone liver transplantation and subsequently developed cardiac deposition of WT TTR (20, 23). Recently, Saelices and colleagues provided direct evidence of amyloid fibrils extracted from heart tissues of patients with ATTR in promoting nucleation of WT and monomeric TTR ex vivo (24).

Considerable work has been done on determining the thermodynamic forces that drive self-assembly of amyloid fibrils as well as the earlier aggregation of misfolded TTR monomer to form intermediates and oligomers (9, 26, 30, 35–38). To further investigate the interactions among TTR assemblies of monomers, dimers, tetramers, and oligomers, we devised a recombinant fusion tag on the protein to artificially induce TTR polymerization via the tag. By adding this induced TTR polymer as a nucleation seed to a mixture of TTR monomer, dimer, and tetramer, we examined the dynamic changes of these TTR forms in response to the seed.

## Results

### Construction of biotin-TTR fusion and SA-induced polymer.

**Figure 1:**
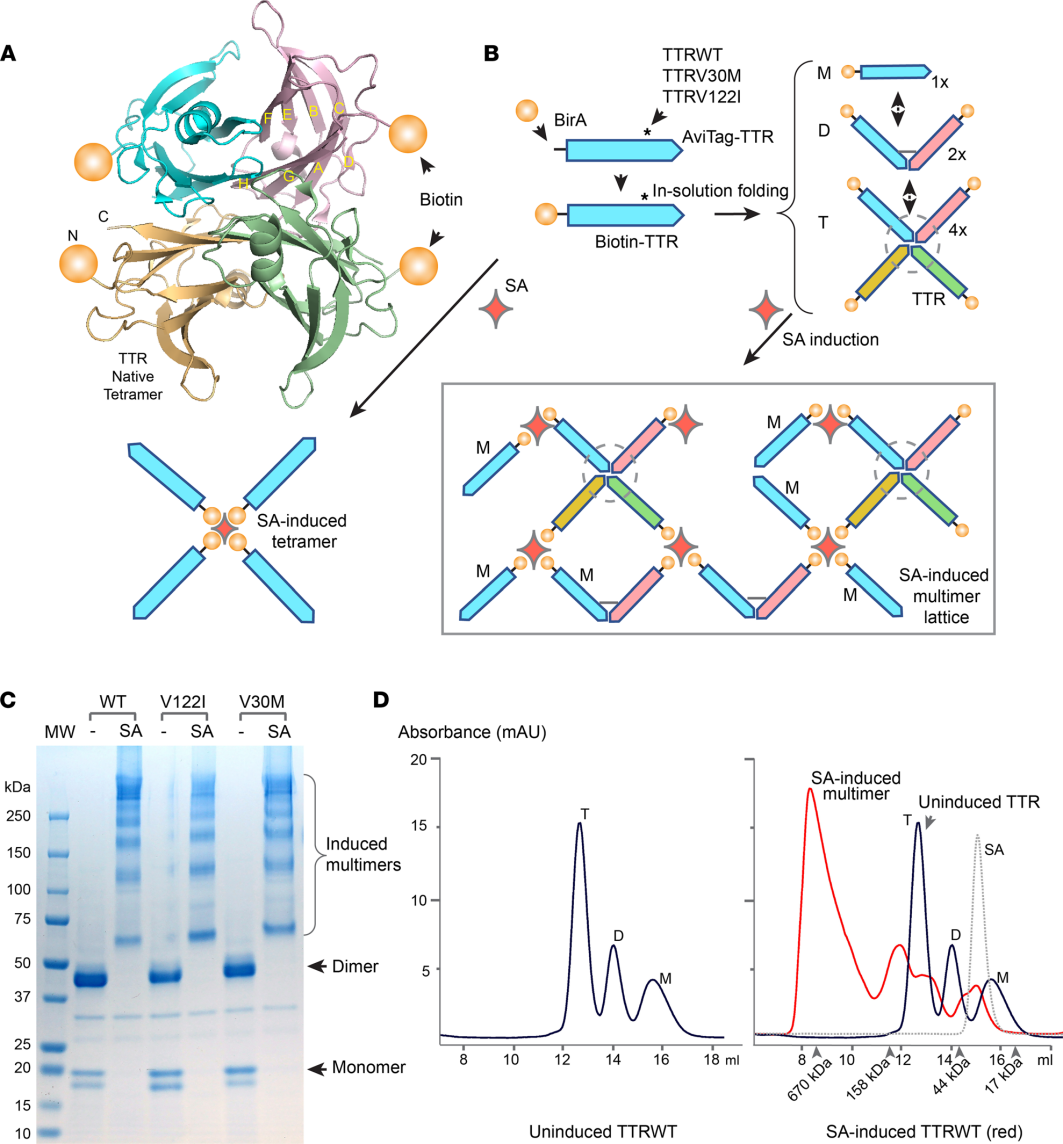
Biotin-TTR fusion and SA-induction model. (**A**) Crystal structure of tetrameric TTR (PDB 5H0V) (58). The 2 sets of antiparallel β-strands that form the β-sheets of DAGH and CBEF were marked in 1 of the subunits (upper-right). Biotin moieties (represented by spheres) were added to the N-termini of all TTR subunits. (**B**) Schematics of site-specific biotinylation by BirA and subsequent multimeric induction by SA. Full-length TTR variants (asterisks) of WT, V30M, and V122I were each fused with an N-terminus AviTag, which was biotinylated. In the presence of SA (lower left: follow arrow), 4 biotin-TTR monomers formed SA-TTR tetramers. In the absence of SA (right panel: follow arrow), soluble TTR formed a mixture of monomer (M), dimer (D), and tetramer (T), in which the broken circle represents the natural tetrameric fold of TTR. Following SA induction of these mixed TTR forms (Bottom right: follow arrow), larger TTR complexes were formed, jointed by monomer, dimer, and tetrameric TTRs (in box). (**C**) WT, V122I, and V30M TTRs in the presence or absence of SA were resolved by reducing SDS PAGE, and subsequently stained with Coomassie blue. Without induction by SA, all variants existed predominantly as SDS-resistant dimers with the presence of less abundant monomers. Following SA induction, TTR formed larger complexes of greater than 600 kDa. (**D**) Biotinylated *TTR*WT in 1M urea pH 7.6 solution formed 3 distinct complexes of M, D, and T as revealed by SEC (left panel). Following induction by SA (right panel), TTR in multimeric complexes with SA gained molecular size (the red line for after induction compared with the solid black line for before induction; SA alone: dotted line). The results of V30M and V122I TTRs with SA induction are in Supplemental Figure 1.

We constructed full-length human *TTR*WT, V30M, and V122I proteins each with an N-terminal AviTag, which was subjected to site-directed biotinylation catalyzed by BirA enzyme (Figure 1, A and B). Via tight binding between the biotin tag and tetrameric SA, larger TTR complexes were formed in conjunction with SA as determined by SDS PAGE (Figure 1C). A TTR-SA lattice structure (modeled in Figure 1B) consisted of 20–40 TTR subunits per induced complex as measured by size-exclusion chromatography (SEC) (Figure 1D and Supplemental Figure 1; supplemental material available online with this article; https://doi.org/10.1172/jci.insight.150131DS1).

### Distinct distributions of TTR monomer, dimer, and tetramer contents among WT, V30M, and V122I at pH 7.6 in 1M urea.

**Figure 2:**
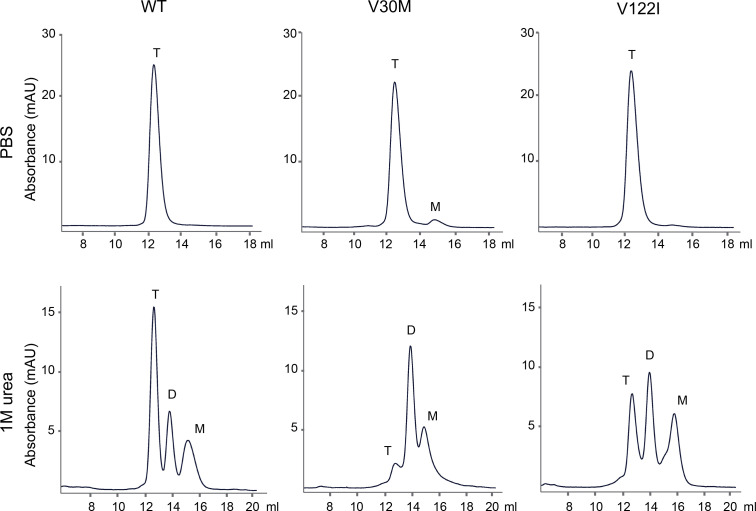
TTR monomer vs. dimer vs. tetramer distribution among WT, V30M, and V122I variants at pH 7.6 in 1M urea. Recombinant TTR proteins were subjected to either PBS at pH 7.4 (top panels) or 1M urea condition at pH 7.6 (bottom panels). SEC analyses showed all variants stayed predominantly as tetramers in PBS, as expected. However, TTR separated into monomer (M), dimer (D), and tetrameric (T) forms under 1M urea condition. In addition, the individual variants of TTRWT, TTRV30M, and TTRV122I showed different distributions of their monomeric, dimeric, and tetrameric contents. All experiments were repeated 3 times.

Without induction by SA, WT, V30M, and V122I appeared as predominantly stable tetramers at pH 7.4 in PBS (Figure 2). As low pH conditions were known to promote disassembly of TTR tetramers into monomers (39, 40), we also characterized the variants at pH 4.4 in either PBS or 1M urea solution. Switching to pH 4.4 caused instant formation of protein precipitation. Over a longer period, there was further increase of turbidity (Supplemental Figure 2), indicating formation of large protein aggregates in keeping with previous reports (40, 41).

Visible protein precipitation usually contains thousands of protein subunits in amorphous aggregates (42, 43). We were more interested in earlier formation of soluble oligomers comprised of only dozens of protein subunits. To this end, we followed an alternative workflow that first involved the denaturing of protein in 8M urea, followed by gradual renaturing through dialysis using 1M urea at pH 7.6. Upon analysis by SEC, *TTR*WT, *TTR*V30M, and *TTR*V122I showed very different distributions among their monomer, dimer, and tetramer contents (Figure 2). *TTR*WT had the highest tetramer level among the variants, with the lowest level of monomers. In contrast, *TTR*V30M dimer formed a tall peak, in keeping with the understanding of V30M’s propensity to form aberrant intermolecular disulfide bridges (13, 44, 45). *TTR*V122I had a relatively “balanced” tetramer, dimer, and monomer distribution, with the highest level of monomeric form compared with its WT and V30M counterparts. These results, despite being obtained from an unnatural process of protein renaturing, were generally in agreement with the expectation of *TTR*WT being most stable, in contrast to *TTR*V30M and *TTR*V122I that showed the propensity of adapting dimeric and dimeric/monomeric amyloidogenic folds, respectively.

### Distinct dynamics in time-dependent oligomeric transformation among TTR variants of WT, V30M, and V122I.

**Figure 3:**
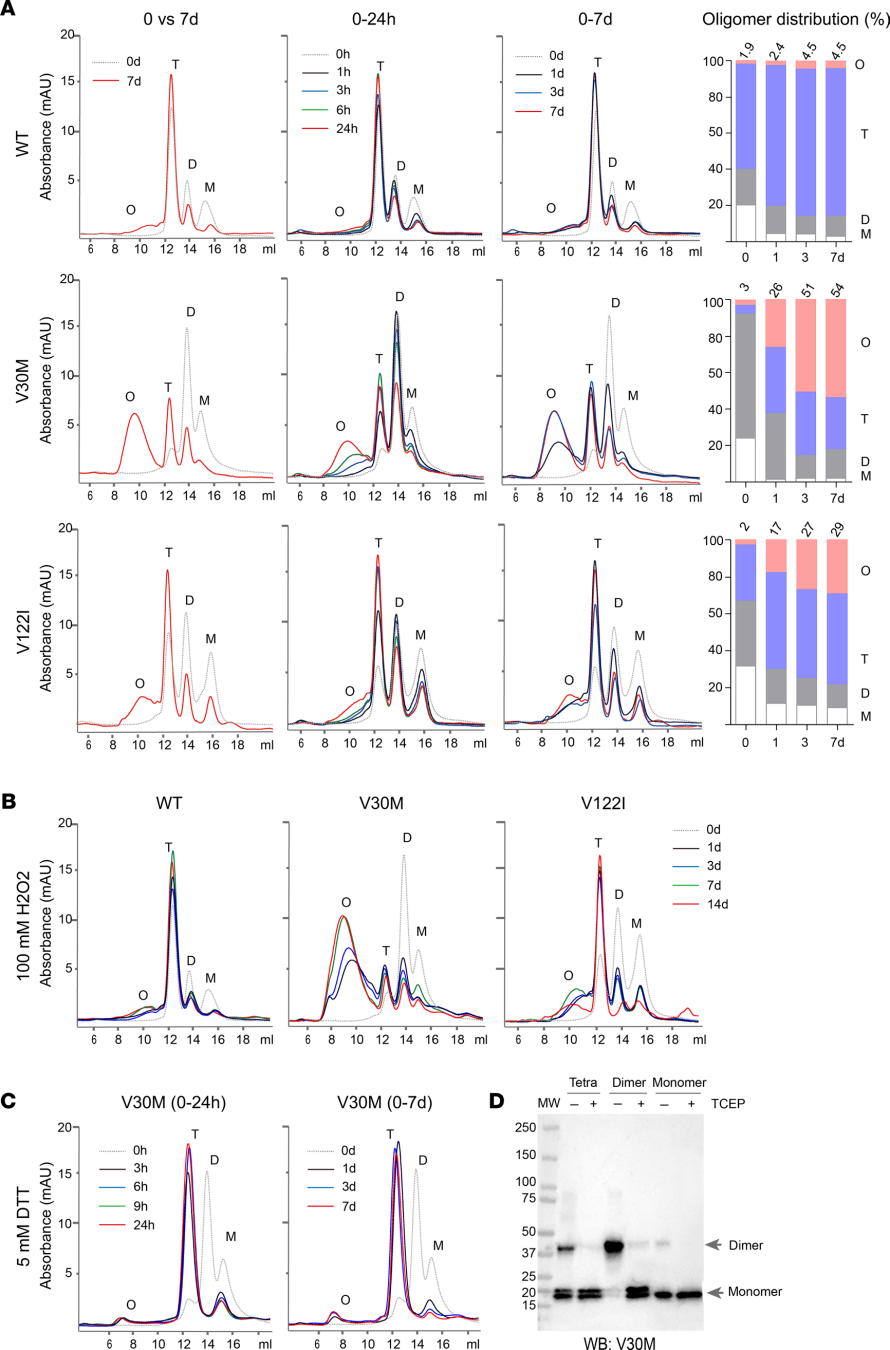
Dynamics in oligomeric transformation among TTR variants of WT, V30M, and V122I. (**A**) Freshly thawed TTR proteins in 1M urea pH 7.6 solution were incubated at room temperature for up to 7 days. In between, aliquots were taken from the reaction at indicated time points of 0, 1, 3, 6, and 24 hours, and 3 and 7 days. Left panels compared SEC profiles between 0 and 7 days in terms of monomeric (M), dimeric (D), tetrameric (T) and oligomeric (O) contents. The right panel bar graphs showed the percentage distribution among M, D, R, and O contents as calculated from AUC of the respective peaks over the 7 day period. (**B**) Treatment of TTR solutions with 100 μM H_2_O_2_ further elevated the levels of O (compare **A** with **B**), particularly in V30M. (**C**) Treatment of *TTR*V30M with 5 mM DTT greatly increased tetramers (compare **B** with **C**). Meanwhile, the D contents completely disappeared. (**D**) Monomeric, dimeric, and tetrameric contents of *TTR*V30M were separated and collected by SEC. The fractions with the presence or absence of TCEP were resolved by SDS PAGE. TTR bands were visualized by IB using anti-TTR antibody. All experiments were repeated 3 times.

Next, we set out to measure early oligomeric transformation of those TTR variants in 1M urea at pH 7.6 by running SEC. Freshly thawed proteins were placed at room temperature on a rotating platform for up to 7 days. Aliquots were collected from the reaction in a time series from 1 hour to 7 days and subsequently analyzed by SEC (Figure 3A). In addition to the 3 distinct peaks in the lower molecular range of monomers, dimers, and tetramers, a UV-absorption reading of protein gradually increased in the higher molecular range between 200 and 600 kDa (Supplemental Figure 3). These intensities reflected the time-dependent accumulation of TTR oligomers in 1M urea solution.

Among the variants, WT TTR had the mildest increase in the amount of oligomers (Figure 3A). It was also noted that the peak heights for WT monomers and dimers gradually decreased, whereas the tetrameric content increased only slightly. It is conceivable that while the tetrameric form of WT TTR was stable, the accumulation of new TTR oligomers was largely assembled from WT monomers and possibly dimers as well. In contrast, *TTR*V30M showed the largest accumulation of its oligomer signals during the same period of time (Figure 3A). Meanwhile, the heights of both dimer and monomer peaks were greatly reduced. Unexpectedly, the tetrameric amount substantially increased, which could only be explained by the contribution of refolding from TTR monomers or dimers, or both concurrently (Figure 3A). *TTR*V122I, which had prominent dimer and monomer peaks at the beginning, showed a modest increase of oligomer levels (comparisons of AUC in Figure 3A) that were greater than that of WT but substantially smaller than that of V30M.

It is interesting to note that in addition to the comparisons of AUC for total oligomer contents, there were clear distinctions in the average molecular size of TTR polymers among the variants (Supplemental Figure 3). *TTR*WT had the smallest average polymer size of approximately 200 kDa, the equivalent of approximately 10 protein subunits; *TTR*V122I had its average polymer size of approximately 300 kDa, the equivalent of approximately 15 protein subunits; whereas *TTR*V30M had its average polymer size of approximately 500 kDa, the equivalent of approximately 25 protein subunits. However, this trend of polymer size was only associated with variant types, regardless of the length of incubation time (Figure 3A), indicating that each variant adapted a distinct assembly unique to the type of mutation and the associated structural fold.

### Oxidative condition further accelerated TTRV30M’s oligomeric transformation.

Next, we examined the effect of 100 μM H_2_O_2_ on these variants in 1M urea at pH 7.6 (Figure 3B). Both *TTR*WT and *TTR*V122I had relatively moderate increases of their oligomeric contents over a period of 14 days compared with the condition without H_2_O_2_ treatment (compare Figure 3, A and B). In contrast, *TTR*V30M had a greater accumulation of its high molecular weight oligomers after being treated with H_2_O_2_ (Figure 3B). There was also a further increase of the average molecular size of V30M oligomer contents over time. These results were largely in agreement with the understanding of *TTR*V30M being able to form an aberrant intermolecular disulfide bridge. Conversely, treatment of *TTR*V30M with reducing agent Dithiothreitol (DTT) at 5 mM in 1M urea and pH 7.6 completely abolished dimeric forms, partially reduced the level of monomers, but greatly restored tetramer content with an increase of more than 10 folds in its level over time (Figure 3C). To further investigate the disulfide bridge formation in *TTR*V30M, we separately isolated SEC fractions of monomers, dimers, and tetramers, and then subjected the fractions to SDS PAGE. Under the nonreducing condition, the V30M dimer appeared to be coupled exclusively by disulfide bridge because Tris (2-carboxyethyl) phosphine (TCEP) converted all dimers to monomers (Figure 3D). In contrast, the tetramers contained only a small number of disulfide-linked dimers in the presence of predominately noncovalently linked subunits (Figure 3D) that were expected to adopt their normal tetrameric fold. These results indicated that the instability of *TTR*V30M was largely driven by aberrant intramolecular disulfide connections that favored a dimeric configuration.

### Artificially induced multimeric seeds capable of depleting TTR monomers and dimers within minutes.

**Figure 4:**
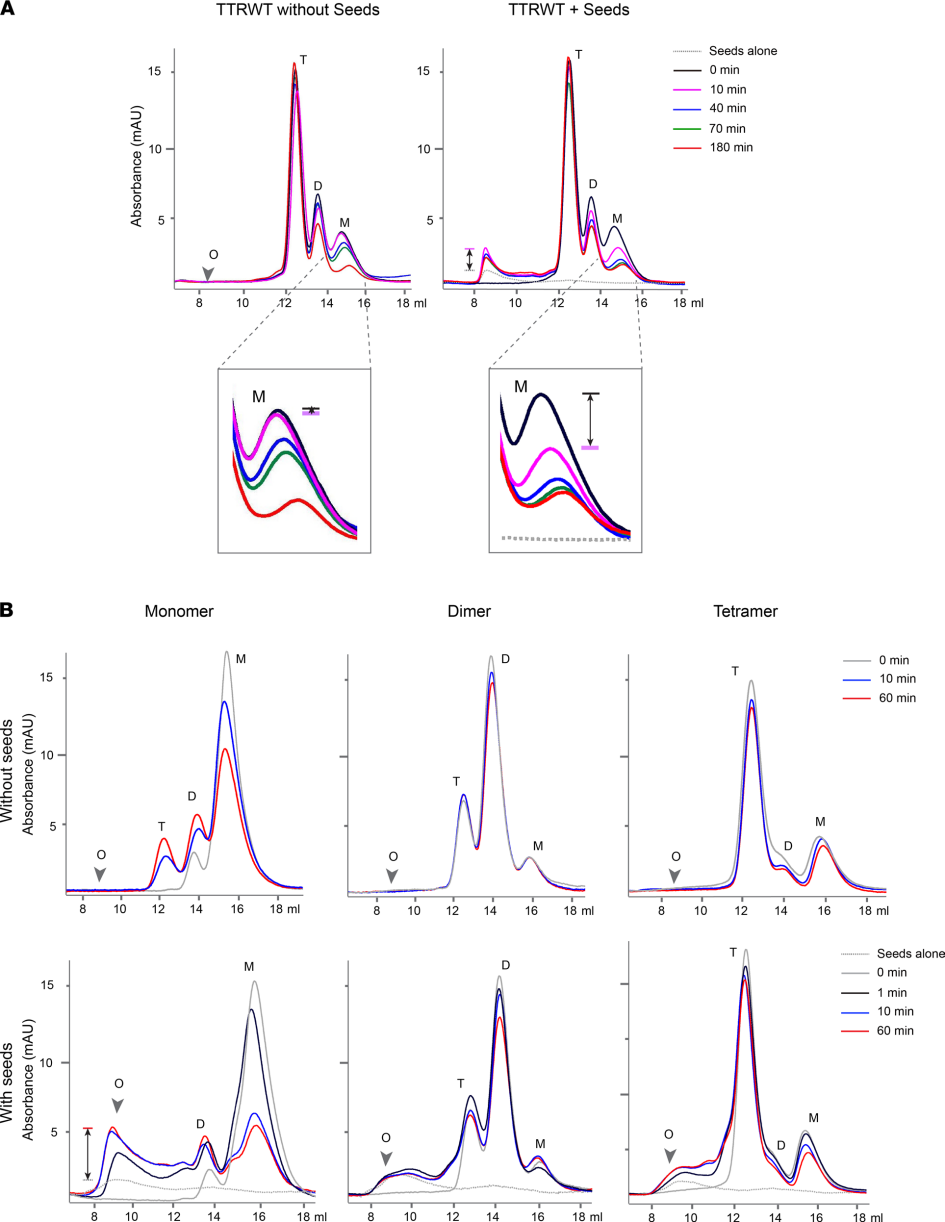
Rapid coaggregation of *TTRWT* monomers by artificially induced SA-TTR complexes. (**A**) *TTR*WT was incubated in the presence or absence of SA-induced *TTR*V30M seeds (right and left panels, respectively; preparation of induced *TTR*V30M seeds is shown in Supplemental Figure 1) in 10:1 w/w ratio for a total of 180 minutes. Aliquots were taken from the reaction at indicated time points and subsequently analyzed by SEC. The seeds-alone sample was separately analyzed by SEC. Monomeric (M) dimeric (D), tetrameric (T), and oligomeric (O) contents are indicated. Insets below show the M peaks in the time series. The arrow shows the range of O increases on top of the seed amount. Additional results of *TTR*WT, *TTR*V30M, and *TTR*V122I with the seeds are in Supplemental Figures 3 and 4. (**B**) Monomeric content of *TTR*WT before induction was purified by SEC and then subjected to conditions with or without the *TTR*V30M seeds (top-left and bottom-left panels, respectively). Aliquots taken from 0, 1 (immediately after the spiking-in of seeds), 10, and 60 minutes were analyzed by SEC. There was the contrasting difference between the presence or absence of the seeds. Without the seeds, there was a gradual shift of monomer contents to newly formed dimers and tetramers. With the seeds, there was a rapid increase of high molecular weight of TTR oligomers (arrow indicates the change in levels). Within minutes, the multimeric seeds depleted most monomers in forming high molecular weight protein complexes. In contrast to *TTR*WT monomers, dimeric and tetrameric fractions did not react to seed-induced aggregation. All experiments were repeated 3 times.

We asked about potential seeding effects on folding energetics that promote TTR oligomeric aggregation. We were particularly interested in whether amyloid seeding can accelerate oligomerization of monomers and dimers. To this end, we added a substoichiometric amount of SA-induced *TTR*V30M multimer as the seed to *TTR*WT in 1M urea buffer. We then measured the changes of TTR complex size in a time series (Figure 4). It should be noted that this SA-induced TTR multimer was not expected to structurally resemble TTR oligomers that adapt β-sheet stacking (15). In theory, our artificial aggregation of TTR through SA-mediated interaction with the N-terminal biotin tag results in a greater number of exposed DAGH sheets available for nucleating soluble TTR monomers (schematics in Figure 1B).

Following the “spike-in” of SA-V30M multimers to freshly thawed *TTR*WT, there was a rapid reduction of the monomer peak (Figure 4A). To a lesser degree, *TTR*WT dimers were also reduced in response to the seeds, whereas the tetramer peak height remained relatively unchanged. Meanwhile, within 10 minutes, the high molecular weight of the approximately 600 kDa content rapidly increased (Figure 4A and Supplemental Figure 4), which was likely attributable to an induced aggregation of monomers and dimers toward the SA-V30M seed. It is important to note that the overall molecular size of seed-induced complexes was larger than that of spontaneously self-aggregated *TTR*V30M (Figure 4A compared with Figure 3A). *TTR*WT in the absence of the seeds showed little increase in oligomers. Instead, the slow reduction of its monomer and dimeric contents resulted in an increase of tetramers, suggesting a process of monomers adapting correct refolding into tetramers (Figure 4A and Supplemental Figure 4). In contrast, in the presence of the artificial inducer of SA-V30M as seeds, TTR monomers rapidly coalesced with the seeds instead of refolding into tetramers (Figure 4A and Supplemental Figure 4).

Next, we focused on the response of *TTR*WT monomers to the seeds in the absence of possible interference by other TTR forms. First, by running SEC, we isolated the monomers and subjected them to conditions either with or without SA-V30M seeds (10% w/w) (Figure 4B). In the absence of the seeds, TTR monomers gradually reduced their levels while there was an increase of both dimeric and tetrameric contents without formation of oligomeric TTR (Figure 4B). In contrast, following the spike-in of the seeds, within the first 10 minutes) there was already a drop of monomer levels with a rapid increase of high molecular weight oligomers (Figure 4B). Meanwhile, there was little change in dimer and tetramer levels, suggesting a potent nucleation ability of the artificial seeds towards TTR monomers. In parallel, we also isolated dimeric and tetrameric *TTR*WT and subjected them to reactions with or without SA-V30M seeds. Unlike the rapid response of monomeric *TTR*WT to seeding, the tetramers and dimers were not affected after multimer seeds were added (Figure 4B). It is also interesting to note that despite the rapid drop of monomer contents after reaction with seeds between 0–10 minutes, the remaining monomer levels between 10–60 minutes changed very little. This suggested the presence of a mixture of misfolded TTR monomers that responded to the seeds and a smaller fraction of stably folded TTR monomers that was resistant to aggregation, which is consistent with the persistence of monomer contents associated with dimer and tetramer fractions.

### New ELISA kit based on the high valency of poly-TTRV30M multimer for detecting misfolded TTR.

**Figure 5:**
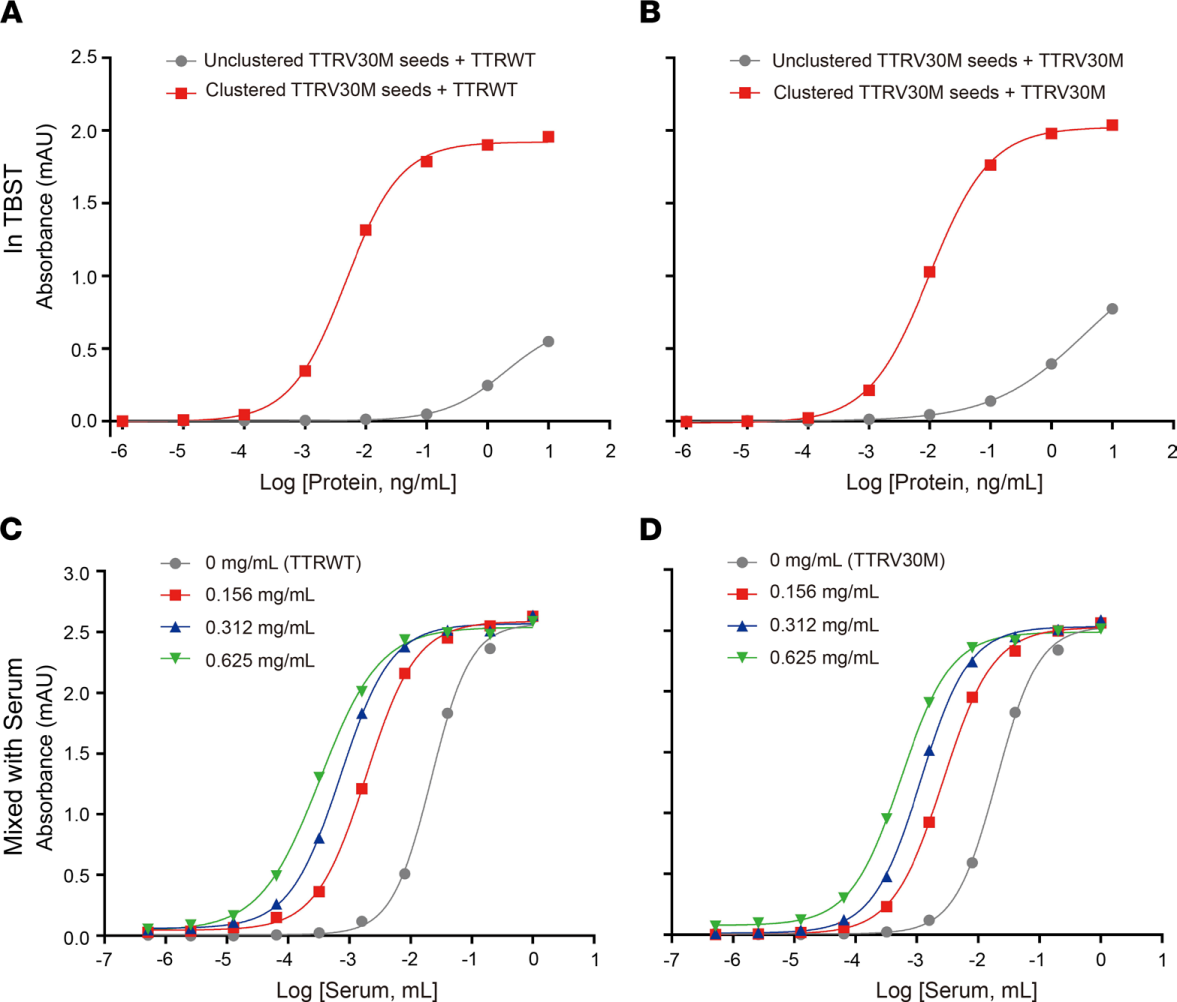
Construction of synthetic poly-*TTR*V30M–based ELISA to detect misfolded TTR contents in serum. Given that SA-clustered synthetic TTR polymer can specifically coalesce monomeric forms of TTR in solution as analyzed by SEC (Figure 4), here we immobilized SA-induced poly-*TTR*V30M seeds on ELISA plates. (**A** and **B**) The coated wells were incubated with various concentrations of either WT (**A**) *TTR*WT with TMT label for detection using anti-TMT antibody; details in Methods) or V30M (**B**) *TTR*V30M with TMT label) recombinant proteins. The control wells contained uninduced *TTR*V30M seeds, which did not present high avidity compared with SA-clustered *TTR*V30M seeds. As expected, wells coated with the clustered *TTR*V30M seeds bound both *TTR*WT and *TTR*V30M. Similarly, for testing whether this ELISA methodology using clustered *TTR*V30M can detect unstable and potentially amyloidogenic TTR species in blood samples, the detection kit was applied to human serum. Whole serum proteins were labeled with TMT, and following different dilutions, the samples were incubated with the ELISA plate. Serum proteins captured by clustered *TTR*V30M on the plate were detected by anti-TMT antibody. (**C** and **D**) As controls, purified recombinant *TTR*WT (**C**) or *TTR*V30M (**D**) (also labeled with TMT) as the spike-in was added to the serum samples at indicated concentrations. The results showed the methodology capable of detecting unstable TTR contents in serum.

Next, we wanted to exploit the high valency of synthetic poly-TTR seeds in selective binding of structurally unstable, and thus potentially amyloidogenic, TTR. Instead of measuring coalescence of TTR by the poly-*TTR*V30M seeds in solution (Figure 4), we immobilized the seeds on ELISA plates as capturing probes (Figure 5), as described in Methods. Separately, we prepared the “prey” proteins of recombinant WT and V30M TTR with Tandem Mass Tags (TMT) to facilitate their detections by an anti-TMT antibody (Thermo Fisher). In contrast to uninduced *TTR*V30M, SA-induced poly-*TTR*V30M–coated wells captured TMT-labeled WT and V30M TTR in TBS Tween (TBST) (Figure 5, A and B). Next, we explored the ELISA kit for detecting serum proteins that can be captured by these poly-*TTR*V30M seeds (Figure 5, C and D). First, we labeled pooled human sera with TMT and divided the sample in a dilution series to be incubated with immobilized seeds. As expected, serum protein signals were detected with the seed-coated ELISA plate. To further ascertain that misfolded TTR in serum can be detected by the method, we spiked-in recombinant *TTR*WT and *TTR*V30M proteins that had been partially destabilized in 1M urea as positive controls. As expected, the ELISA kit could detect the spiked-in TTR controls (Figure 5, C and D). These results provided the initial validation of poly-*TTR*V30M seeds-based methodology for measuring potentially amyloidogenic TTR contents in serum, although we caution that further characterization of the ELISA kit is needed by using confirmed ATTR samples.

### Intravenously injected SA-TTR multimer-formed renal deposits in mice.

**Figure 6:**
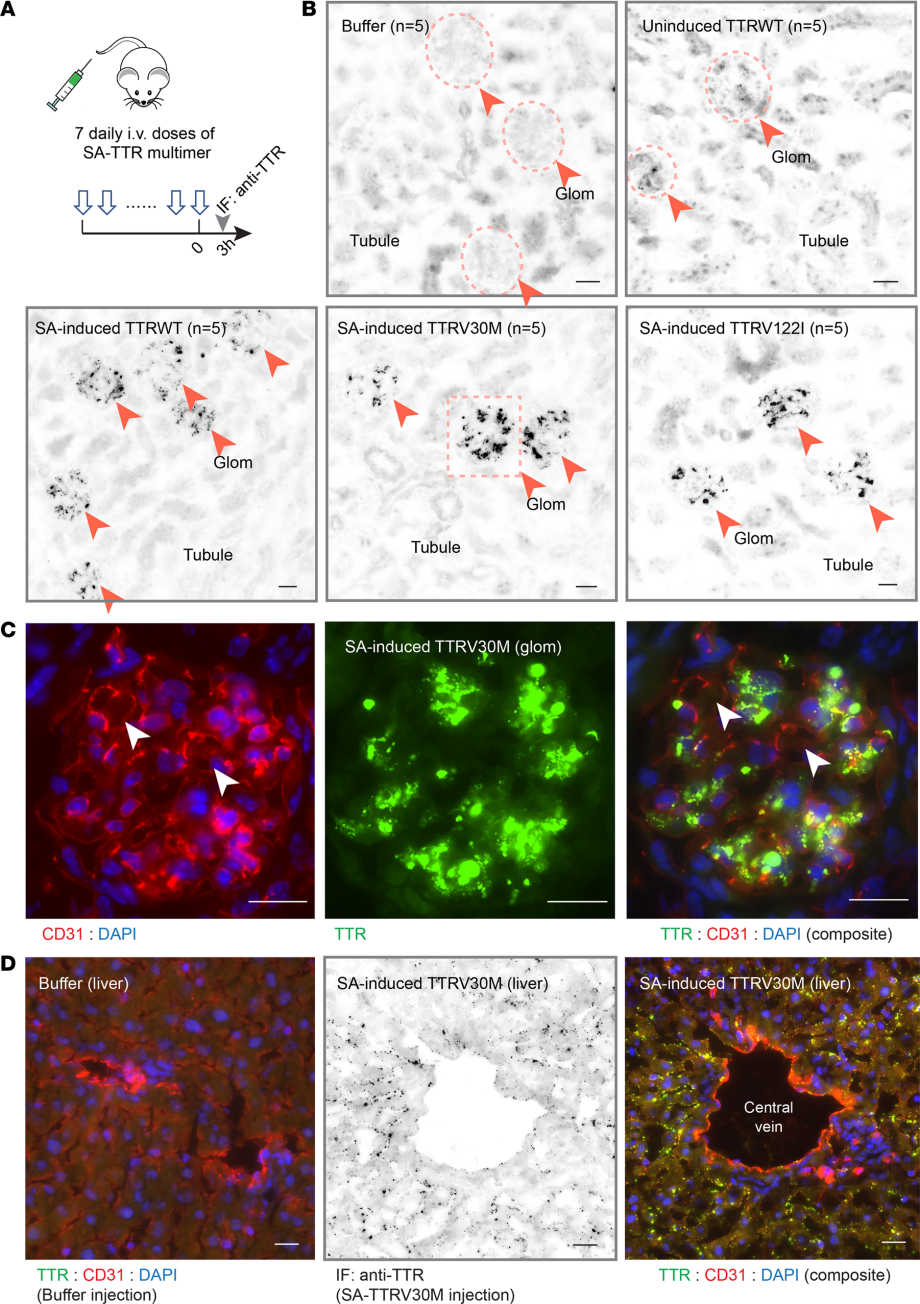
SA-TTR multimer formed renal deposits in mice. (**A**) A total of 25 mice (*n* = 5/group) received 7 i.v. doses of buffer, uninduced *TTR*WT, or SA-induced *TTR*WT, *TTR*V30M, or *TTR*V122I at 15 mg/kg BW per dose for 7 consecutive days (arrows). The mice were harvested (arrowhead) 3 hours after the last injection on day 7. Kidney, liver, and heart specimens were stained with anti-TTR antibody by IF. (**B**) IF images of the kidney sections showed no TTR signal from the buffer and only a trace amount of uninduced TTR in some, but not all, glomeruli (top panels). In contrast, all SA-induced TTR variants in their multimeric forms showed prominent TTR signals as puncta of varying sizes exclusively in the glomerulus (arrowheads and circled areas). All glomeruli were stained positive, whereas renal tubules only showed background-level signals with no TTR puncta (bottom panels). (**C**) Inset of SA-induced V30M image from bottom center panel in the boxed area of **A**. (Images from induced *TTR*WT and *TTR*V122I injections are in Supplemental Figure 5). CD31 marked glomerular capillaries. Cross sections of the capillary loops are pointed by arrowheads (left panel of CD31 staining). Strong SA-TTR deposition was observed with aggregates varying in size and shape (middle panel). Composite image of the glomerulus showed TTR deposits were not in the capillary lumen (arrowheads). Instead, the deposits were located to the glomerulus mesangial areas. (**D**) The liver of SA-V30M–injected mice showed scattered TTR puncta that also appeared to be outside of vascular lumina (marked with CD31). Scale bar: 30 μm.

Considering the nucleation capability of this SA-induction model, we sought to determine possible in vivo effects of the induced complexes. To this end, we directly injected SA-induced multimers in mice at 15 mg/kg BW (Figure 6A). Each group was assigned 5 mice to receive 7 daily injections of *TTR*WT, *TTR*V30M, or *TTR*V122I. Organs such as the heart, the liver, and the kidney were harvested 3 hours after the last injection and tissue specimens were probed for TTR using immunofluorescence (IF). Few tissue deposits were formed by uninduced recombinant TTRs (Figure 6B and Supplemental Figure 5). In contrast, prominent TTR deposition was observed in the kidney glomerulus of all mice that received the injection of SA-induced TTRs (Figure 6B and Supplemental Table 1). Costaining of the specimens of vascular endothelium marker CD31 showed TTR deposits outside of the capillary lumen (Figure 6C). It is, therefore, conceivable that the deposits were formed after TTR had exited blood circulation and entered glomerular mesangial areas in the kidney. Furthermore, TTR puncta in the kidney appeared to vary greatly in size and shape (Figure 6, B and C), possibly attributable to further growth of TTR aggregates in renal interstitium. Transmission electron microscopy (TEM) images of the glomerulus revealed electron-dense amorphous deposits in the mesangium (Supplemental Figure 6), indicating the formation of nonfibrillar oligomeric intermediates. In the liver, SA-induced *TTR*V30M also formed deposits (Figure 6D), which were consistent with the general function of the liver in clearing large protein complexes from circulation.

### Intravenously injected SA-TTRV30M multimer-formed prominent cardiac deposits in mice.

**Figure 7:**
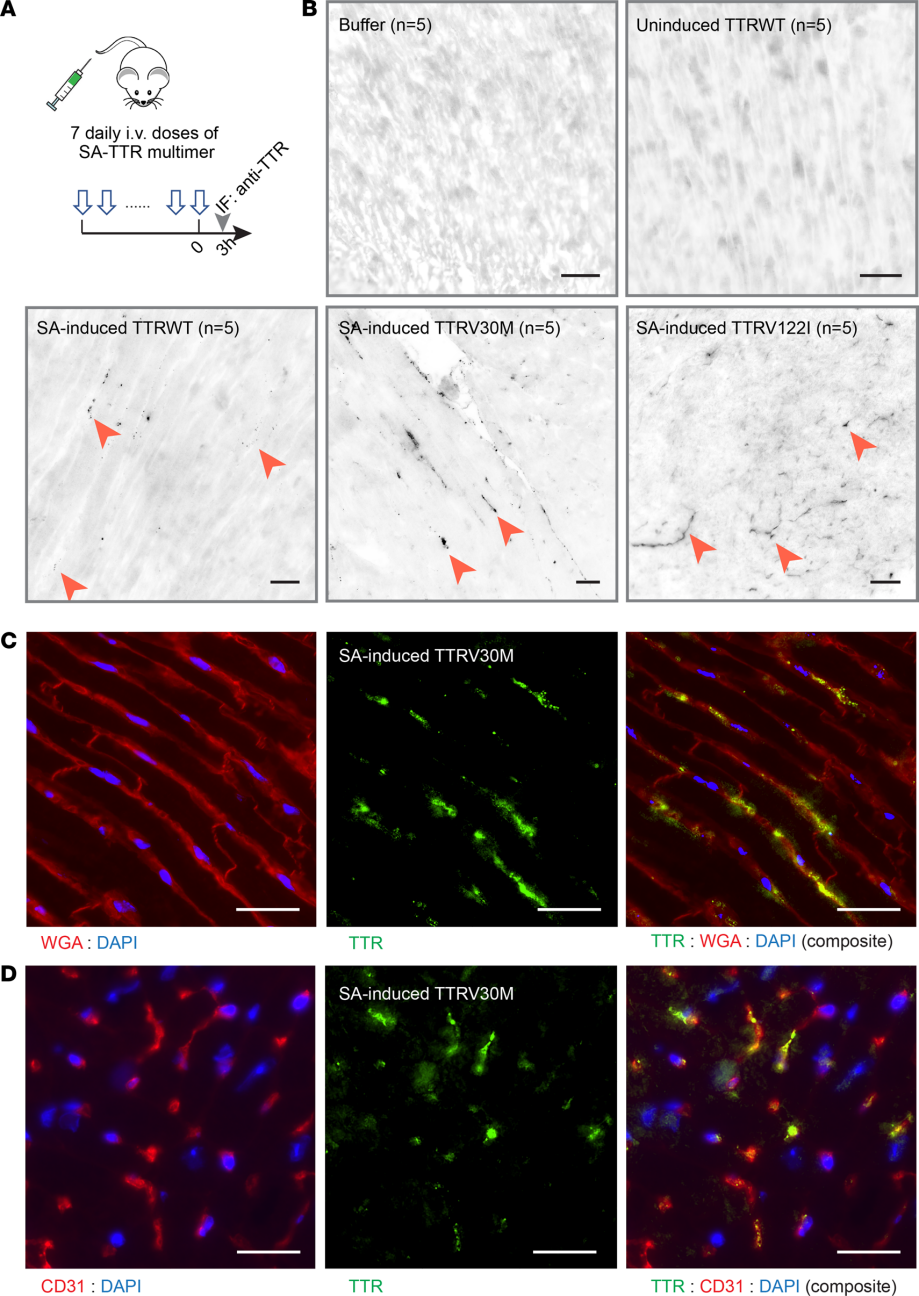
SA-TTR multimer formed cardiac deposits following i.v. injection in mice. (**A**) Mice (*n* = 5/group) from the same injection series as in Figure 6 were collected for histologic analysis of the heart. (**B**) IF staining of TTR was performed to the heart specimens. Representative images are shown for each group. SA-induced groups showed positive staining of TTR deposits (black dots: arrowheads pointed). Additional images of V30M deposition are in Supplemental Figure 6. (**C** and **D**) Mice injected with SA-induced *TTR*V30M showed cardiac TTR deposits. The deposits partially overlapped with WGA and CD31 staining signals of cardiac sarcolemma in **C** and vasculature in **D**. Scale bar: 50 μm.

In contrast to the kidney that developed deposits from SA induction regardless of the type of TTR variants, some, but not all, mice in each injection group had cardiac deposits after 7 daily doses of the injection (Figure 7A and Supplemental Table 1). There were also far fewer TTR deposits in the heart in terms of fluorescence intensity and prevalence. Among the 3 variants induced with SA, *TTR*V30M had by far the strongest fluorescence intensity and a broader presence of deposition in the heart, whereas WT and V122I deposits were sparse (Figure 7B).

From *TTR*V30M injection, most deposits were in the myocardium layer (Supplemental Figure 7), reminiscent of chronic deposition in ATTR-CM. Longitudinal sections showed the distribution of *TTR*V30M deposits located primarily in the intermyofibrillar space along the sarcolemma (Figure 7C). Cross sections showed the deposits associated with myocardial vessels (Figure 7D), likely in the interstitial space between the sarcolemma and the capillary. Overall, these TTR deposits varied in size and fluorescence intensity in highly clustered patterns (Figure 7 and Supplemental Figure 7). It should be noted that 4 weeks after the last injection, TTR deposits were no longer detectable in the heart or in the kidney (Supplemental Figure 8).

## Discussion

In this work, we focused on comparing the dynamics of TTR transitions among monomeric, dimeric, tetrameric, and early intermediates and polymeric states. The study was performed in the context of common TTR variants of WT, V30M, and V122I in the presence or absence of a synthetic *TTR*V30M multimer as the seed for nucleating TTR. The key innovation of the study was the construction of TTR with an N-terminus biotin tag that, through interacting with SA, robustly assembled TTR into 20–40 subunit lattices. This induced *TTR*V30M multimer lattice functioned as a high avidity trap to recruit TTR monomers and dimers within minutes, while more stable TTR tetramers were unaffected by the multimeric trap. Our observations were consistent with what Saelices et al. previously showed using ATTR fibrils extracted from patients’ heart tissues to accelerate monomeric TTR aggregation ex vivo (24). In keeping with the observed activity of our synthetic TTR multimer to specifically coalesce misfolded monomers, immobilized synthetic multimers devised as the capture reagent in an ELISA kit were used to measure serum contents of misfolded TTR species. In addition, our artificially induced *TTR*V30M multimer also demonstrated an in vivo capability of forming ATTR-like cardiac deposits in mice that possibly resemble TTR seeding in early stages of ATTR clinical development. To our limited knowledge, our system was the first cardiac model of acute ATTR deposition. Compared with the transgenic mouse method (46, 47) that takes almost 2 years to develop cardiac deposits, our injection model can quickly generate the phenotype with an extensive display of ATTR-like deposits in the myocardium that likely resemble early events of amyloidosis.

Extraction of TTR fibrils from ATTR hearts inevitably contains a large amount of non-TTR proteins and requires healthy tissue controls (24, 48). In contrast, our synthetic TTR multimers from recombinant production were more homogenous. As measured by size-exclusion chromatography, more than 90% of TTRs were assembled into higher-order multimers following SA induction (Figure 1D and Supplemental Figure 1). We should emphasize that these SA-TTR multimers were assembled from a mixture of TTR monomers, dimers, and tetramers. In conjunction with the tetrameric state of SA, SA-TTR complexes were expected to adapt a lattice-like configuration (Figure 1B), consisting of an estimated 20–40 TTR subunits as determined by the combined sizes of the complexes (Figure 1D). Structural analysis by Saelices et al. specifically identified β-strands that are more exposed in TTR monomers than in tetramers to explain how monomers are prone to primary nucleation (49). In our SA-TTR lattices, TTR proteins were in spatial vicinity to each other without adapting a cross-β spine configuration of mature amyloid fibril. It is conceivable that the high density of adhesive surfaces presented by the cluster of individual monomers and dimers within the SA-TTR complex could rapidly nucleate soluble monomers due to an avidity effect. Although amyloid deposits in tissues are predominantly bundled fibrils, each in a zipper spine configuration, the combined fibril ends may still present an avidity advantage to recruit soluble TTR monomers. In amorphous amyloid aggregates, which the artificial SA-TTR complexes may resemble, the ability to recruit soluble TTR might also be attributable to presumed high avidities. A prior study by Saelices et al. (49) showed strong seeding effects at physiological pH 7.6 of either cardiac fibrils or in vitro constructed monomeric TTR (“MTTR”) aggregates that Jiang et al. first introduced (50).

It is of particular note that TTR oligomerization is a complex and still partially understood process and may even follow distinct assembly pathways. Pathogenic mutations are structural factors for aberrant fibrillar assembly that can cross-seed WT TTR. Environmental conditions can also influence the conformational flexibility of TTR monomers in forming morphologically different oligomers (26, 36, 51). TTR oligomerization is driven by monomer misfolding and monomer-monomer interactions via solvent-exposed proamyloidogenic surfaces (50). Nevertheless, oligomerization is a dynamic process of complex monomer-monomer, monomer-oligomer, and oligomer-oligomer interactions, which also involve adaptive conformational changes of the terminal TTR subunits in growing prefibrillar aggregates. Furthermore, tissue tropism and cytotoxicity also contribute to the ultimate formation of mature amyloid fibrils in disease.

Again, it should be clarified that the 1M urea condition at pH 7.6 by no means resembles the natural environment. We favored the condition only because it greatly eases the transformation among monomeric, dimeric, tetrameric, and oligomeric forms, whereas PBS at pH 7.6 stabilizes tetramers (Figure 2) and a low pH condition causes quick disassembling of tetramers and amyloid fibril formation (39, 40). Indeed, among *TTR*WT, *TTR*V30M, and *TTR*V122I, the 1M urea condition showed clear distinctions among the variants in terms of the distribution of monomer, dimer, and tetramer contents (Figure 2). Therefore, the 1M urea condition that seemed to amplify the destabilizing effects was ideal for our analytical purposes.

Unlike the endogenous process, our workflow of renaturing TTR variants into their folded states greatly boosted the aberrant monomer and dimer contents. In human cells, the endoplasmic reticulum-Golgi not only has chaperones to assist protein folding, but also has intrinsic quality control steps to further prevent the secretion of misfolded TTR (52–54). The actual levels of misfolded TTR relative to the levels of properly folded tetramers in blood are expected to be very low, even in patients with ATTR. The slow progressive nature of the disease in patients and in mouse models has greatly limited research advancement. Our recombinant multimers represent an attractive alternative in which phenotypes of TTR deposition in the heart can be achieved in days by injecting artificially aggregated SA-TTR complexes. However, it should be clarified that the organ deposits of our induced TTR multimers did not resemble natural amyloid fibrils of cross–β-sheet assembly. Our ex vivo results showed a strong propensity of the artificial complex to nucleate TTR monomer, a characteristic shared with ATTR oligomeric precursors. However, although our synthetic high-order TTR seeds could rapidly nucleate misfolded monomers, there was only a modest increase of thioflavin T (ThT) signals (Supplemental Figure 9), indicating the assembly of TTR subunits within the oligomeric TTR lattice was not in the form of long amyloid fibrils. This is consistent with the lack of green birefringence signals from Congo red staining of the heart and the kidney under polarized light (not shown). Future studies need to address the long-term consequence of whether the artificial SA-TTR complex can cross-seed the nucleation of endogenous TTR in vivo.

## Methods

### Construction of recombinant TTR with AviTag.

Full-length human TTR (Uniprot, P02766) was used as the template for constructing recombinant *TTR*WT protein. Encoding cDNA was synthesized by Integrated DNA Technologies with codons optimized for *E*. *coli* expression. This TTR cDNA was fused to an N-terminus AviTag sequence (encoding amino acids GLNDIFEAQKIEWHE). The fusion was cloned into a PET30a vector (Invitrogen) with the addition of a 6xHis purification tag. *TTR*V30M and *TTR*V122I variants were made by mutagenesis to the WT template.

### Recombinant protein production and purification.

Recombinant TTR expression was induced with 0.3 mM isopropyl-β-d-thiogalactoside for 16 hours at 25°C. Bacterial pellets were collected by centrifugation and then stored at –80°C. On the day of purification, the bacterial pellet was resuspended in 0.13M NaCl, 20 mM Na_2_HPO_4_ buffer (pH = 7.4) supplemented with 0.5 mg/mL lysozyme for 30 minutes followed by sonication; the mixture was then subjected to centrifugation. The clear supernatant that contained AviTag-TTR was loaded onto a Histrap column (GE Healthcare), and the recombinant protein was collected with elusion buffer containing 250 mM imidazole. Imidazole was then removed by desalting and buffer exchange, adjusted to PBS pH 7.4. The final protein concentration was calculated using a BCA kit (Pierce) and the protein purity was determined by SDS-PAGE analysis. TTR in 1M urea from protein renaturing treatment was prepared from the inclusion body. After lysis, the bacterial pellets were collected and then washed in 1M urea with PBS (pH 7.6). The resulting pellets were lysed and resuspended in 8M urea buffer (pH 7.8) at 4°C for 24 hours. Following an additional centrifugation, supernatant was loaded onto a Histrap column. Denatured TTR was collected following elution using imidazole. Renaturing was performed by dialysis with 1M urea pH 7.6 overnight. Biotinylation and SA-induced multimerization were performed in the 1M urea buffer.

### Site-directed biotinylation of TTR to an N-terminus AviTag and multimeric induction of biotin-TTR with SA.

BirA biotin ligase was produced using a BL21 (DE3) expression system. Purified AviTag-TTR proteins were subjected to site-directed biotinylation in 0.2 mM ATP, 5 μM MgCl2 together with BirA (55). Each TTR polypeptide was labeled with 1 biotin group attached to the AviTag sequence. To induce multimerization of biotin-conjugated TTR, SA (Agilent Technologies) was added at approximately a 1:4 molar ratio. Unincorporated free biotin was removed by desalting column (GE Healthcare).

### Size-exclusion chromatography and gel analyses of TTR multimericity.

Preparational and analytical size-exclusion chromatography was performed on Superdex 200 Increase 10/300 GL (GE Healthcare). Time series analyses were conducted by running sample aliquots on the same column in succession approximately 30 minutes apart. Gel analyses for estimating the molecular size of TTR complexes were performed by comparing reducing versus nonreducing conditions with or without 50 mM TCEP, respectively. It should be noted that biotin-SA interactions can withstand 1% SDS in sample buffer for SDS PAGE. Reducing condition in the presence of 50 mM TCEP disrupts biotin-SA interactions as well as disulfide bonds between TTR subunits. ThT assay was performed in FLUOTRAC 96-well microtiter plates in 25 μM ThT in phosphate saline and 1 mg/mL of uninduced TTR with or without 0.1 mg/mL of streptavidin-induced (SA-induced) *TTR*V30M seeds at 37°C. Fluorescence signals at 450 nm excitation/485 nm emission were read at indicated time points. For measuring solution turbidity, WT, V30M, and V122I samples at 1 mg/mL concentration were stored in PBS or 1M urea buffer. In a 1:1 ratio (v/v), the samples were then mixed with pH 4.4 solution of 100 mM acetate sodium, 100 mM KCl, and 1 mM EDTA. Turbidity was measured at 405 nm over 96 hours at 25°C. All experiments were repeated 3 times.

### TTR seeding using SA-induced TTRV30M on TTRWT.

Multimers of SA-induced *TTR*V30M seeds were prepared by collecting SEC elution fractions between 8–10 mL of the SA-*TTR*V30M reaction. The seed concentration was then adjusted to 0.1–0.12 mg/mL. The seeds were mixed with recombinant *TTR*WT (1 mg/mL in 1M urea buffer) or its monomer/dimer/tetramer isolates in a 1:10 (w/w) ratio (56). Aliquots were analyzed on the same column approximately 30 minutes apart in succession. All experiments were repeated 3 times.

### Construction of poly-TTRV30M–based ELISA and assay development for detecting serum contents of unstable TTR.

Coating of the microtiter plates was performed with 100 μL of either *TTR*V30M recombinant protein or SA-induced *TTR*V30M multimers at 5 μg/mL concentration in each well at 4°C overnight. Following washing with TBST, the plates were blocked using 1% BSA. For labeling either purified TTR proteins or whole serum with TMT (Thermo Fisher), 800 μg of recombinant protein or 100 μL of serum was desalted (using Pierce desalting spin column) and then incubated with 400 μg TMTzero reagent for 3 hours at room temperature following manufacturer’s standard protocol. The reaction was quenched by the addition of Tris buffer, followed by desalting. Then, in a dilution series, 100 μL of TMT-labeled samples was incubated in the precoated wells for 1 hour at room temperature. Following additional washing steps with TBST, anti-TMT monoclonal detection antibody (Thermo Fisher, 25D5) at 1:1000 dilution and HRP-conjugated anti-mouse IgG secondary antibody were used to detect levels of proteins coaggregated with the multimer TTR bait.

### Injection of recombinant TTR complexes in mice.

BALB/cJ strain of mice (Charles River Labs) between 16–20 weeks of age were separated into groups with an approximate matching distribution of sex and weight. For 7 consecutive days 300 μg of TTR protein was injected via the tail vein. The animals were then sacrificed and the organs, including the heart, the kidney and the liver, were collected 3 hours after receiving the last dose of injection. The specimens were minced into smaller pieces to be embedded in OCT (Thermo Fisher). Frozen tissues were sectioned at 4 μm thickness for staining using anti-TTR antibody (Agilent Technologies, A0002) at 1:200 dilution, and goat anti-rabbit IgG Alexa-488 (Novus Biologicals, A11034) at 1:400. Counterstains used anti-mouse CD31 antibody (BD Pharmingen), wheat germ agglutinin (WGA) Alexa Fluor conjugate (Thermo Fisher) at 1:100 dilution, and DAPI (Sigma-Aldrich). The method for transmission electron microscopy of kidney deposits was described previously (57).

### Study approval.

All animal studies were carried out in accordance with regulations of the NIH for the care and use of laboratory animals (Northwestern University IACUC protocol: IS00009990, OLAW: A3283-01).

## Author contributions

LG and JJ conceived of the presented idea. LG developed the study and performed the experiments. XX and PL assisted with the experiments. LG and JJ performed the analysis, drafted the manuscript, and assembled the figures. All authors discussed the results and commented on the manuscript.

## Supplementary Material

Supplemental data

## Version 1 04/08/2022

Electronic publication
