# Supplementary material for: High-avidity binding drives nucleation of amyloidogenic transthyretin monomer
Source: JCI Insight. 2022 Apr 8;7(7):e150131. doi: 10.1172/jci.insight.150131 (PMC9057628; doi:10.1172/jci.insight.150131)
Supplement: Supplemental data [file jciinsight-7-150131-s039.pdf]

High-Avidity Binding Drives Nucleation of Amyloidogenic Transthyretin Monomer

Li Gao, Xinfang Xie, Pan Liu, and Jing Jin[*]

corresponding author Jing Jin
Email: jing.jin@northwestern.edu

This PDF file includes:

Supplementary table S1

Supplementary figures S1 to S9

Supplementary table S1. Summary of mouse phenotypes following injection of streptavidin-induced TTR multimer.

Experimental group	Number of mice	TTR deposition in kidney	Average renal TTR score by IF	TTR deposition in heart	Average heart TTR score by IF
*TTR*WT+SA	5	5/5	+++	2/5	+
*TTR*V122I+SA	5	5/5	+++	2/5	++
*TTR*V30M+SA	5	5/5	+++	2/5	+++
*TTR*WT	5	1/5	+	0/5	—
PBS+1M Urea	5	0/5	—	0/5	—

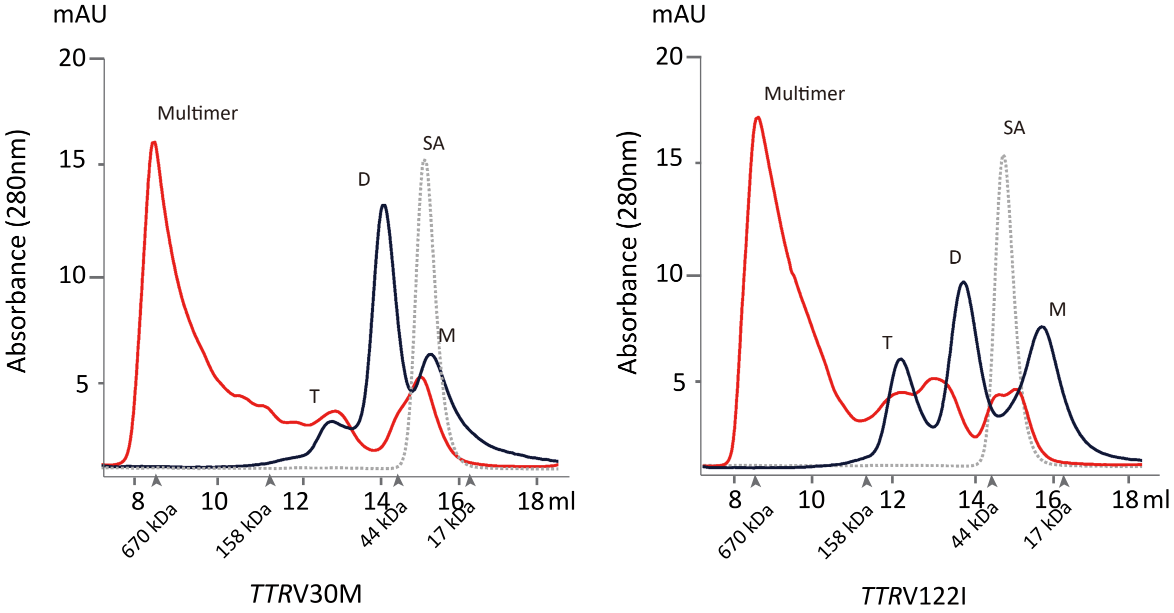

Supplementary figure S1. Biotinylated TTR variants of V122I and V30M were induced for multimerization by streptavidin (SA).

Uninduced TTRs had mono- (M), di- (D) and tetrameric (T) contents as shown by SEC (black lines). Following induction with SA (as shown in Fig1D), the combined SA-TTR multimeric complexes had increased molecular sizes of ~670 kDa (red lines). SA alone was represented by the dotted lines.

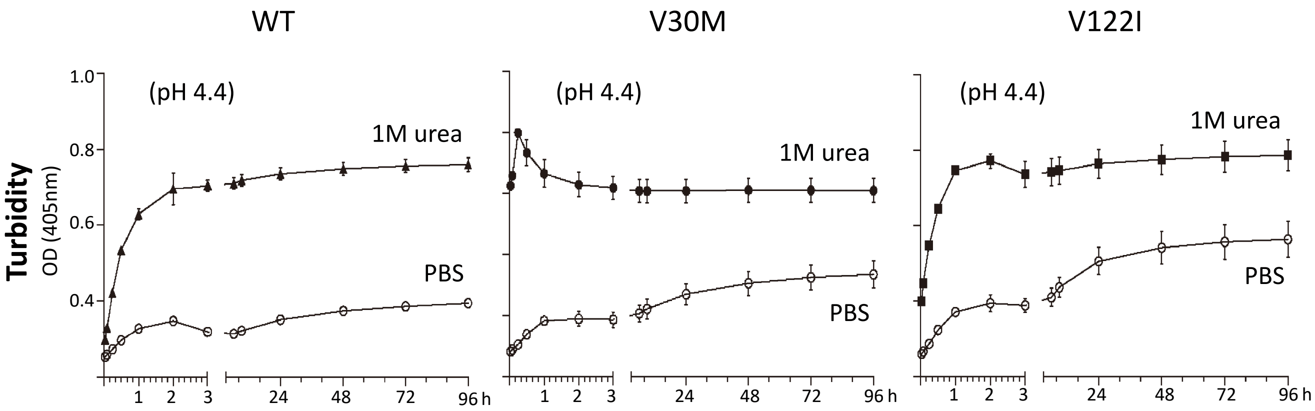

Supplementary figure S2. TTR variants of WT, V30M and V122I were prone to aggregate under low pH. Recombinant TTR proteins in either 1M urea or PBS solution were subjected to pH 4.4 acetate buffer. Solution turbidity was monitored over a period of 96 hours.

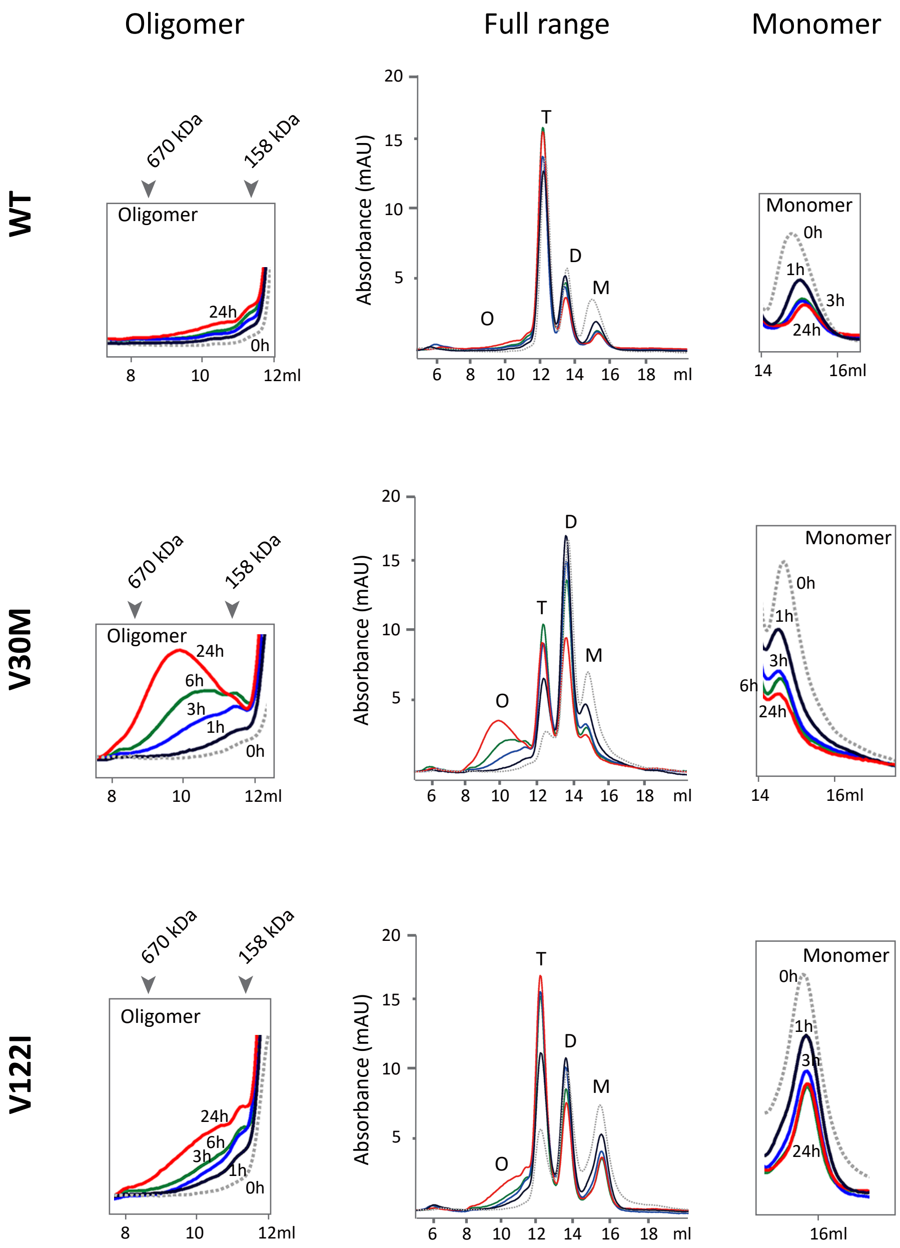

Supplementary figure S3. Dynamic changes of oligomer and monomer contents of TTR variants. Detailed view of Fig3A for within a 24 hour period. Changes in oligomer and monomer contents are shown in insets (left and right panels, respectively).

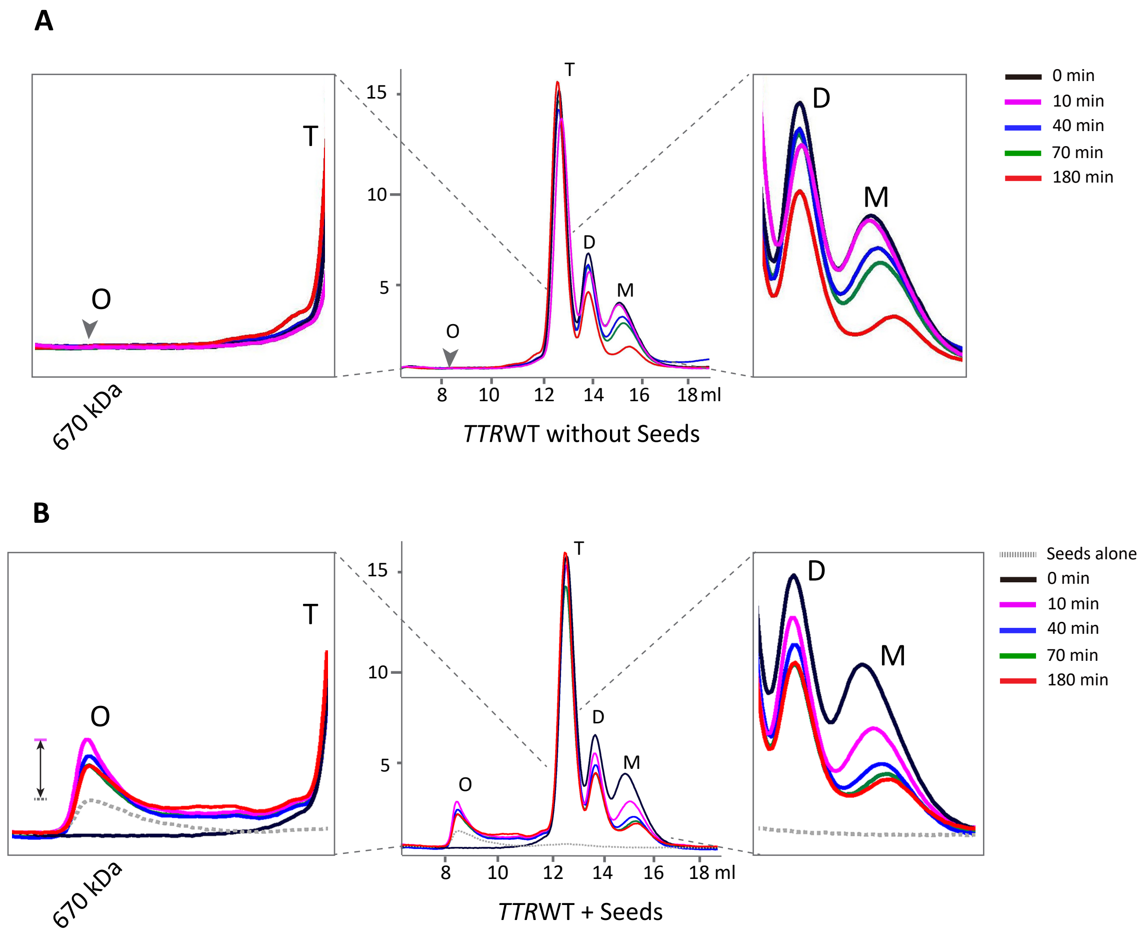

Supplementary figure S4. Derived from Fig4A with insets showing details

Left and right panels in **A** and **B** show selected regions of SEC graph of oligomers (O, left panels) and dimer (D) and monomers (M) (right panels). In **B** with the spike-in of V30M seeds (10% ratio), there was a gradual accumulation of oligomeric contents (Left panel, follow arrow).

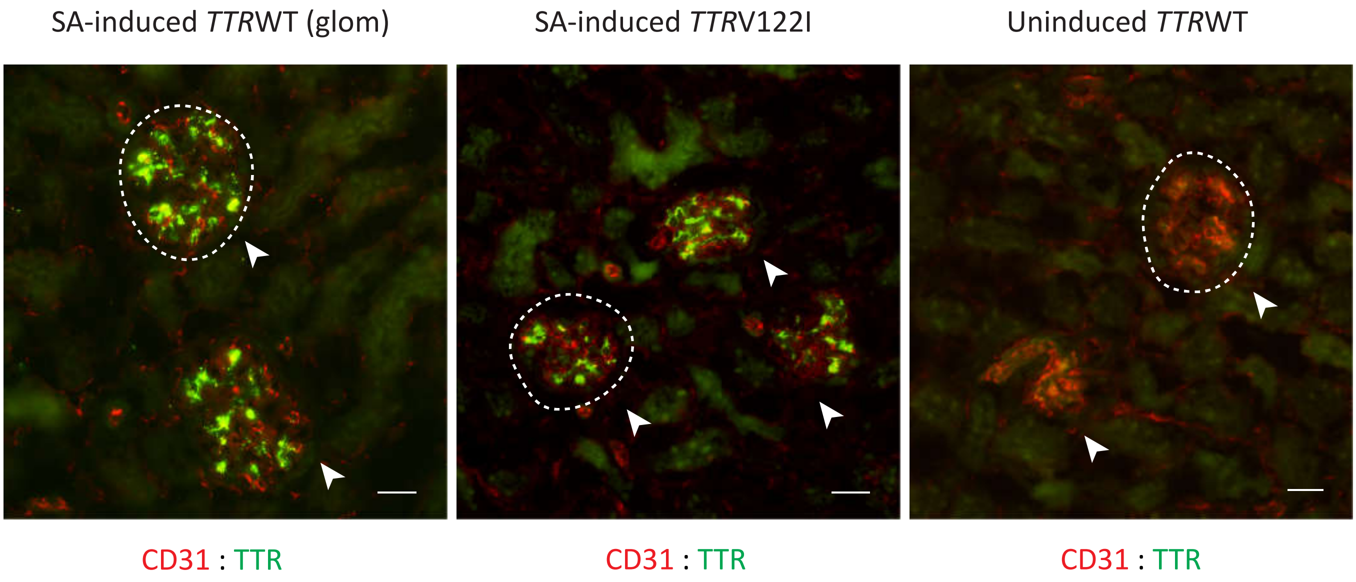

Supplementary figure S5. Renal deposition of streptavidin-induced *TTR*WT and *TTR*V122I complexes. Similar to the experiment shown in Fig6, mice were intravenously injected daily with SA-induced *TTR*WT, *TTR*V122I, or uninduced *TTR*WT as control for seven consecutive days. Kidneys were harvested 3 hours after the last injection and specimens were stained with anti-TTR antibody with anti-CD31 as counterstain. Both SA-induced proteins showed prominent glomerular (circles and arrowheads) deposition, similar to the results from SA-V30M injection (Fig6). In contrast, uninduced *TTR*WT formed only trace deposits in the kidney. Scale bar: 25 µm.

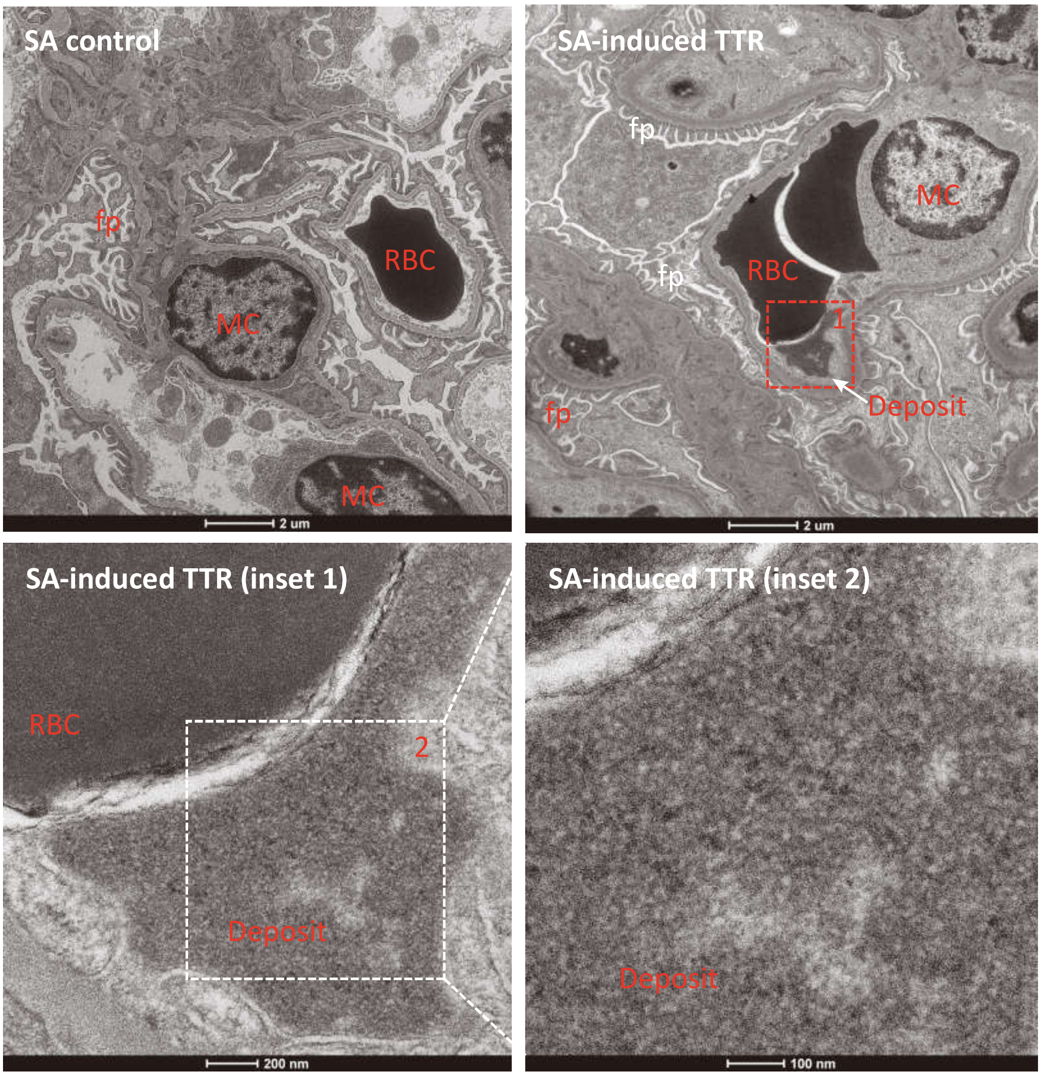

Supplementary figure S6. Transmission electron micrographs of glomerular TTR deposits from injection. Injection of streptavidin (SA: control) did not form kidney deposits in the glomerulus (upper left; MC: mesangial cell; fp: foot processes; RBC: red blood cell). Meanwhile, i.v. injection of SA-induced TTR oligomer complexes had led to deposition of electron-dense materials in the glomerulus (upper right; box 1: pointed by arrow). Lower panels show the electron-dense deposit at higher magnifications.

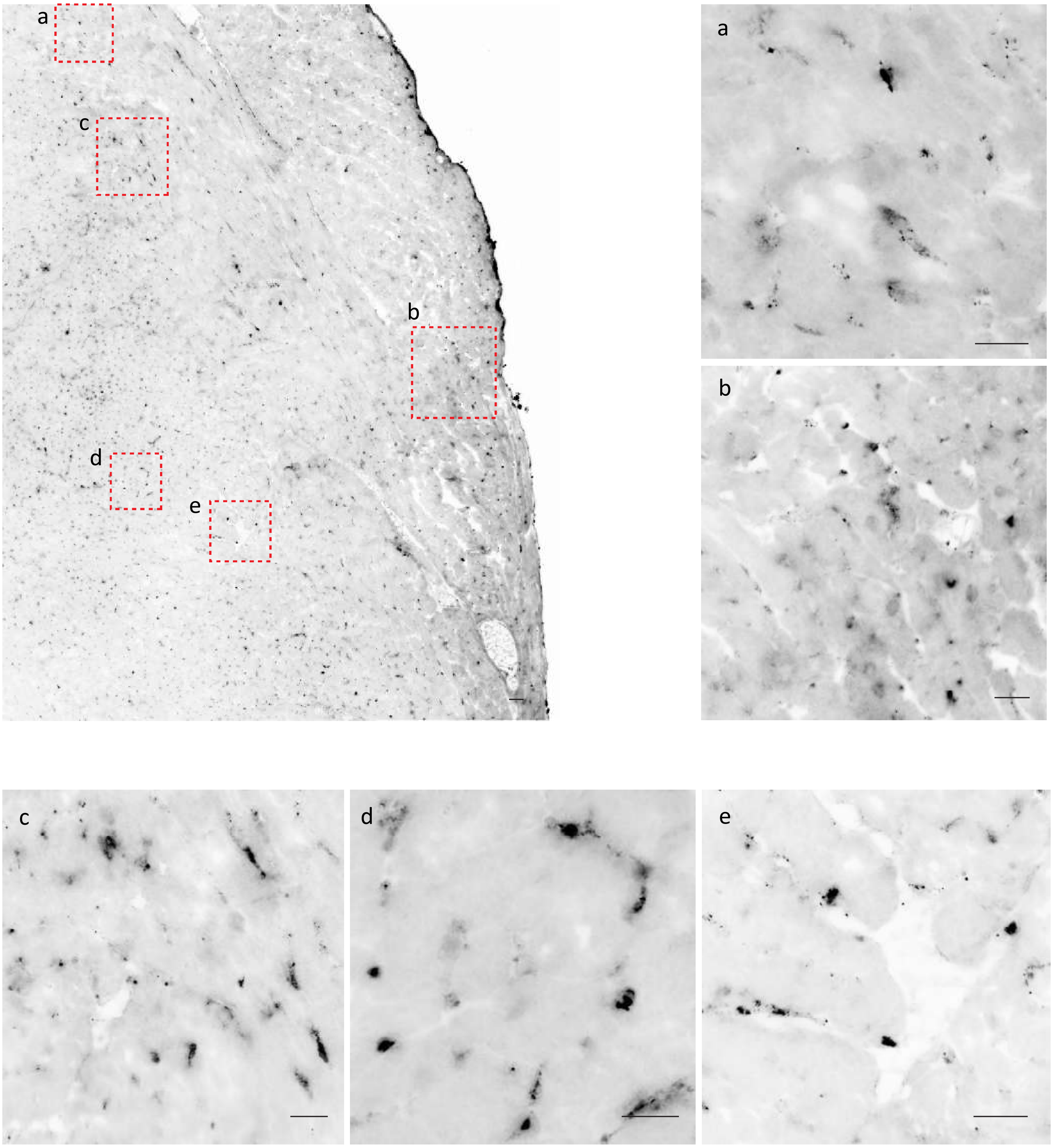

Supplementary figure S7. Cardiac deposits of SA-induced *TTR*V30M (an overview of Fig7).
Mouse injected with SA-induced *TTR*V30M. Heart specimen was stained with anti-TTR antibody. Upper left: a broad view of anti-TTR immunofluorescence image of the heart. Insets a-e show selected areas (red boxes) at higher magnifications of TTR deposits in the myocardium. Scale bar: 50µm.

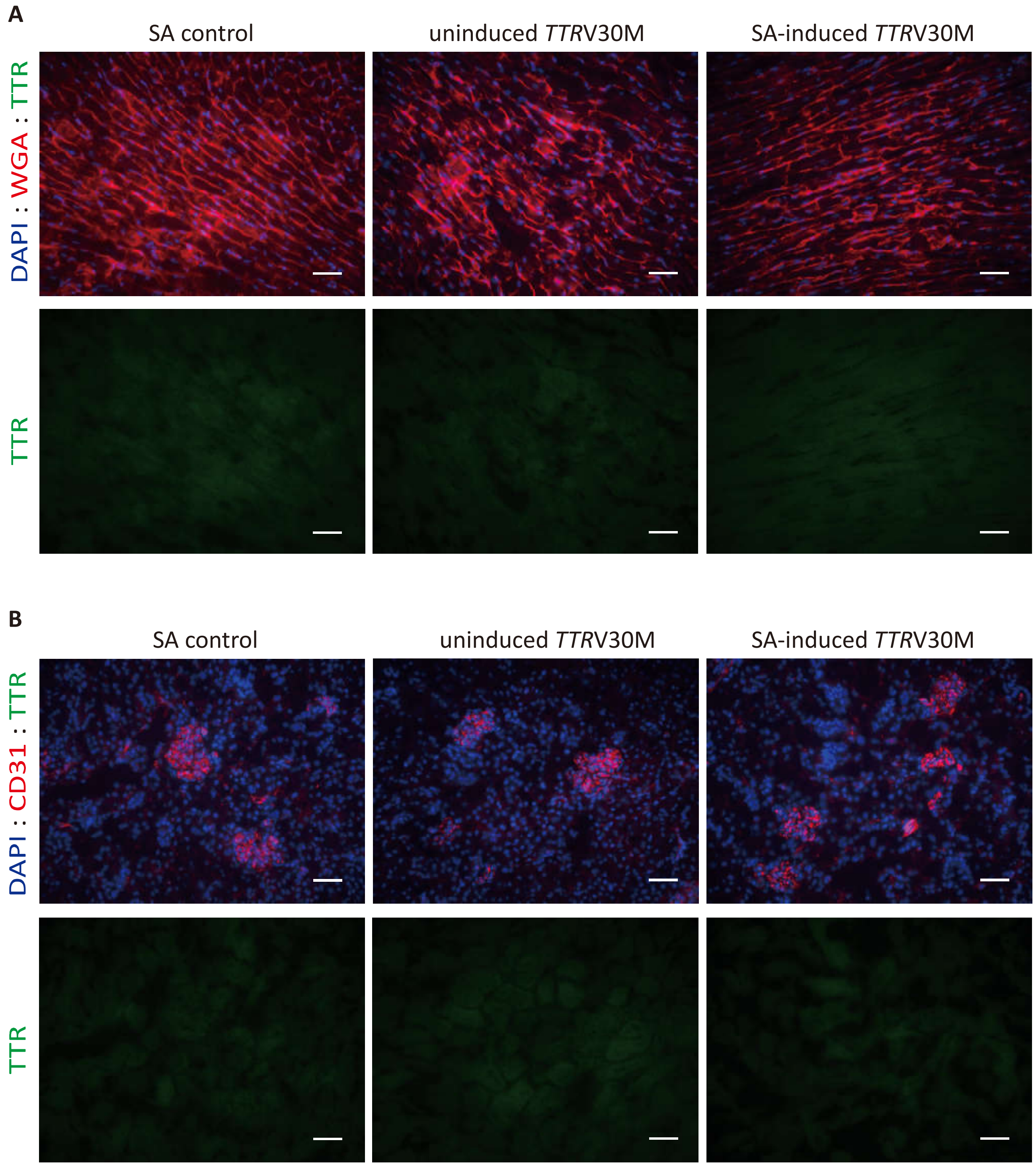

Supplementary figure S8. Streptavidin-induced TTR deposits in the heart and in the kidney completely disappeared after 4 weeks (as compared to Fig6 and Fig7).
Mice (n=3 in each group) were injected daily with either SA control, uninduced recombinant *TTR*V30M, or SA-induced recombinant *TTR*V30M oligomers for 7 consecutive days. Four weeks after the last injection, the hearts and the kidneys were collected for tripple stainings with anti-TTR, WGA and DAPI for the heart specimens (**A**), and with anti-TTR, anti-CD31 and DAPI for the kidney (**B**). Representative immunofluorescence images are shown. No TTR deposit signals (in green) were observed in the intermyofibrillar space of the heart (marked by WGA) and the glomerulus of the kidney (follow CD31 staining of the glomerular capillary).
Scale bar: 25µm.

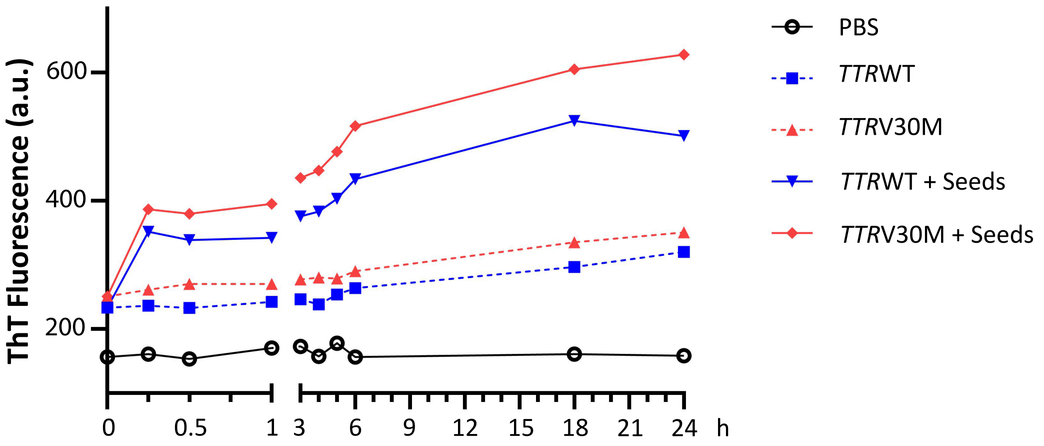

Supplementary figure S9. A modest increase of thioflavin T (ThT) signals with recombinant TTR following using with synthetic oligomeric *TTR*V30M.

In a 10:1 (w/w) ratio, 1 mg/ml of either *TTR*WT or *TTR*V30M (mixtures of monomers and tetramers) were added to microtiter plates with the presence or absence of 0.1 mg/ml synthetic *TTR*V30M oligomeric seeds from streptavidin induction. With 25 µM ThT in the reactions under shaking, ThT fluorescence signals at 450 nm excitation/485 nm emission were read at indicated time points. There was a modest increase in the signals following the addition of synthetic poly-*TTR*V30M seeds, occuring more rapidly within the first 0.25 hour.
